# Unique depot formed by an oil based vaccine facilitates active antigen uptake and provides effective tumour control

**DOI:** 10.1186/s12929-018-0413-9

**Published:** 2018-01-27

**Authors:** Kimberly D. Brewer, Genevieve M. Weir, Iulia Dude, Christa Davis, Cathryn Parsons, Andrea Penwell, Rajkannan Rajagopalan, Leeladhar Sammatur, Chris V. Bowen, Marianne M. Stanford

**Affiliations:** 1 Biomedical Translational Imaging Centre (BIOTIC), Halifax, NS Canada; 2 Department of Diagnostic Radiology, Dalhousie University, Halifax, NS Canada; 3 Department of Physics and Atmospheric Science, Dalhousie University, Halifax, NS Canada; 4 Department of Microbiology and Immunology, Dalhousie University, Halifax, NS Canada; 5 School of Biomedical Engineering, Dalhousie University, Halifax, NS Canada; 6 Immunovaccine Inc., Halifax, NS Canada

**Keywords:** Magnetic resonance imaging (MRI), Cancer, Vaccines, Emulsion, DepoVax, Biomarker

## Abstract

**Background:**

Oil emulsions are commonly used as vaccine delivery platforms to facilitate slow release of antigen by forming a depot at the injection site. Antigen is trapped in the aqueous phase and as the emulsion degrades in vivo the antigen is passively released. DepoVax™ is a unique oil based delivery system that directly suspends the vaccine components in the oil diluent that forces immune cells to actively take up components from the formulation in the absence of passive release. The aim of this study was to use magnetic resonance imaging (MRI) with additional biological markers to evaluate and understand differences in clearance between several different delivery systems used in peptide-based cancer vaccines.

**Methods:**

C57BL/6 mice were implanted with a cervical cancer model and vaccinated 5 days post-implant with either DepoVax (DPX), a water-in-oil emulsion (w/o), a squalene oil-in-water emulsion (squal o/w) or a saponin/liposome emulsion (sap/lip) containing iron oxide-labeled targeted antigen. MRI was then used to monitor antigen clearance, the site of injection, tumour and inguinal lymph node volumes and other gross anatomical changes. HLA-A2 transgenic mice were also vaccinated to evaluate immune responses of human directed peptides.

**Results:**

We demonstrated differences in antigen clearance between DPX and w/o both in regard to how quickly the antigen was cleared and the pattern in which it was cleared. We also found differences in lymph node responses between DPX and both squal o/w and sap/lip.

**Conclusions:**

These studies underline the unique mechanism of action of this clinical stage vaccine delivery system.

**Electronic supplementary material:**

The online version of this article (10.1186/s12929-018-0413-9) contains supplementary material, which is available to authorized users.

## Background

Therapeutic cancer vaccines are often evaluated based on their ability to generate antigen-specific T cell responses, on the assumption that robust T cell responses are required for clinical benefit [1, 2]. In theory, cytotoxic T cells specific to tumour antigens can infiltrate tumours and destroy tumour cells. To ensure continued tumour infiltration by these T cells and objective clinical responses, a robust and sustained circulating T cell response is likely a critical factor. Most vaccine formulations currently used for therapeutic cancer vaccines were adapted from technologies developed for the prophylaxis of infectious disease, and thus may not be able to induce the type of T cell response that is predicted to be effective in oncotherapy [3]. Therefore, novel methods to deliver these unique vaccines to the immune system may be a key enabling technology for this field.

T cell activating vaccines often contain minimal (8–11-mer) peptide antigens designed to be preferentially presented by class I HLA molecules to CD8^+^ T cells. Longer (15–30-mer) peptides may also be included to stimulate a CD4^+^ T cell response, which has been shown to help sustain CD8^+^ T cell responses [4]. As short peptides are generally not immunogenic, immune stimulating adjuvants are included in these vaccine formulations.

Depot formation is also used to further promote immune responses to peptide antigens by protecting them from degradation, prolonging exposure to the immune system and enhancing antigen uptake by antigen presenting cells (APCs) [5]. Classical depot-forming vaccines, such as water-in-oil emulsions, have been shown to enhance induction of CD8^+^ T cells due to slower release of antigen at the injection site [6]. Emulsions prepared with incomplete Freund’s adjuvant (IFA) oil are commonly utilized, yet have had limited success in inducing effective T cell responses in clinical trials [4, 7, 8]. This may be due in part to the inadvertent sequestering of activated T cells to the site of injection, preventing migration to tumours [9, 10]. Water-in-oil emulsions can also be associated with significant local reactogenicity, inducing inflammatory reactions, ulcers and granulomas at the injection site that is often associated with the volume of vaccine administered [11, 12]. Although there have been many significant improvements to the identification and optimization of immunogenic T cell activating peptide antigens, there are still comparatively few options for immune targeting delivery systems that have advanced clinically. Currently, most include variations of emulsions, such as oil-in-water or oil-in-water-in-oil, or use of liposomes [13].

DepoVax™ (DPX) is a unique oil-based formulation that does not require creation of an emulsion, as the formulation has no aqueous component. When formulated with peptide antigens, the DPX formulation can result in robust and persistent T cell immune responses [14, 15]. DPX may also include an adjuvant to help initiate and direct immune responses. To prepare DPX formulations, antigens and adjuvants are prepared in liposomes which are then lyophilized. The resulting cake is reconstituted directly in oil, such as Montanide ISA51 VG, prior to injection. The presence of the lipids ensures that all components of the formulation are suspended in the oil. Preclinical evaluation of DPX formulated vaccines have shown they are effective in controlling tumour growth when administered in a therapeutic regimen [16, 17]. Antigen specific T cells induced by these vaccines can be detected in the circulation and within the tumour microenvironment [18]. The active immune responses induced by DPX can be further enhanced in combinations with other forms of immune therapy, such as metronomic cyclophosphamide and anti-PD-1 [17, 18]. A DPX-based immunotherapeutic designated DPX-Survivac was tested in a Phase 1 clinical study of advanced ovarian patients and induced remarkably high levels of circulating tumour antigen-specific T cells [14, 15]. These observations suggest that DPX-based vaccines can elicit a systemic antigen-specific immune response that can effectively control tumour growth.

Previously, we used magnetic resonance imaging (MRI) to evaluate the biodistribution and clearance of an iron-labeled antigen formulated in DPX [19]. The study demonstrated that peak antigen removal from the site of injection occurs within 3 weeks after immunization and continues for at least 3 three additional weeks in tumour-bearing mice. Antigens accumulated within the vaccine draining lymph node, indicating that this process could be mediated by antigen presenting cells actively taking up the vaccine components in the absence of passive antigen release at the site. We were able to correlate immune responses with the volume of the vaccine draining lymph node relative to the volume of the contralateral tumour draining lymph node. This metric is a viable biomarker that could be useful for clinical trials investigating vaccine therapy, and could assist in distinguishing pseudo-progression from actual progression.

In this study we have used this MRI tracking technique to further evaluate the unique mechanism of action of a DepoVax-formulated vaccine in comparison to classic depot forming vaccines.

## Methods

### Mice

C57BL/6 female mice (4–6 weeks old, pathogen free) were obtained from Charles River Laboratories (St. Constant, PQ). HLA-A2.1/ HLA-DR1 transgenic, β2m^−/−^/ H2D^b−/−^, IAα^−/−^/ IAβ^−/−^/ IEβ^−/−^ knockout mice were obtained from Charles River Laboratories (France) and bred in house. All mice were housed with food and water *ad libitum* under filter top conditions. Experiments involving the use of mice were carried out in accordance with protocols approved by the University Committee on Laboratory Animals at Dalhousie University, Halifax, N.S., Canada.

### Cell lines

The C3 cell line (obtained from Dr. Martin Kast) [20, 21] was maintained in Iscove Modified Dulbecco’s Medium (IMDM; Sigma, St. Louis, MO) supplemented with 10% heat-inactivated fetal calf serum (Sigma, St. Louis, MO), 2 mM L-glutamine (Gibco, Burlington, ON), 50 mM 2-mercaptoethanol (Gibco, Burlington, ON), 100 U/ml penicillin and 100 μg/ml streptomycin (Gibco, Burlington, ON) and grown at 37 °C and 5% CO_2_. Primary immune cells were maintained in RPMI-1640 medium supplemented with 10% heat-inactivated fetal bovine serum (Sigma, St. Louis, MO), 2 mM L-glutamine (Gibco, Burlington, ON), 50 mM 2-mercaptoethanol (Gibco, Burlington, ON), 100 U/ml penicillin and 100 μg/ml streptomycin (Gibco, Burlington, ON).

### Peptides

All peptides were synthesized by PolyPeptide Group at > 90% purity. The H2Db restricted epitope HPV16 E7_49–57_ (RAHYNIVTF; R9F) and the tetanus toxin universal T-helper peptide TT_947–967_ (FNNFTVSFWLRVPKVSASHLE; F21E), were used in vaccine formulations. DPX-Survivac contained the following MHC class I peptides (epitopes from the survivin protein): SurA1.T (FTELTLGEF), SurA2.M (LMLGEFLKL), SurA3.K (RISTFKNWPK), SurA24 (STFKNWPFL), and SurB7 (LPPAWQPFL), and the tetanus toxin universal T-helper peptide TT_830–843_(AQYIKANSKFIGITEL; A16L).

### Vaccine formulations

Vaccines were prepared either as DPX formulation [17, 19], w/o emulsion [22], squalene oil-in-water formulation [23], or saponin/liposome formulation [24]. For DPX, a lipid-mixture containing DOPC and cholesterol in a 10:1 ratio (w:w) (Lipoid GmBH, Germany), R9F, F21E, and a proprietary polynucleotide based adjuvant were formulated in 40% tert-butanol, freeze-dried and resuspended in Montanide® ISA 51 VG (SEPPIC, France). W/o emulsions were prepared by mixing R9F and F21E in sterile water, followed by mixing the prepared antigen solution with equal volume of Montanide ISA 51 VG to form a homogeneous emulsion. Squalene oil-in-water emulsion was prepared by high pressure homogenization of a mixture of Tween80 (Sigma-Aldrich, USA), Span85 (Sigma, USA) and Squalene (Sigma, USA) in Sodium citrate buffer to achieve a particle size < 150 nm and then mixed with equal volume of sterile water containing R9F and F21E. For Saponin/liposomes formulation, Saponin liposomes were prepared first by freeze-drying (Virtis, SP industries, USA) a mixture of DOPC (Lipoid GmBH, Germany), Cholesterol (Solvay Pharmaceuticals, Belgium) and 3D–MPL (Avanti polar lipids, USA). The freeze-dried lipid mixture was reconstituted with QS21 Saponin (Sigma, USA) prepared in sodium phosphate buffered saline to form liposomes and then extruded to achieve a particle size < 150 nm. The prepared sized Saponin/liposomes formulation was then mixed with an equal volume of sterile water containing R9F and F21E. All vaccines delivered 5 μg of R9F and 5 μg of F21E per dose. In MRI experiments only, i.e. tumour challenge studies comparing DPX-R9F to water-in-oil emulsion-R9F (*n* = 52) and DPX-R9F to other vaccines (*n* = 15), R9F was labeled with superparamagnetic iron oxide (SPIO) prepared as in [19].

Vaccines for the in vitro release study were prepared either as a DPX formulation [10, 17–19], a w/o emulsion, or an aqueous control (AC) using the DPX-Survivac peptides. For DPX-Survivac, the peptides and a polynucleotide adjuvant were formulated with a DOPC-Cholesterol mixture (10:1 w:w) in 0.1 M sodium acetate, freeze-dried, and then reconstituted in Montanide ISA 51 VG. The w/o formulation (w/o-Survivac) was prepared by dissolving the peptides and polynucleotide adjuvant in 0.1 M sodium acetate, followed by mixing the prepared antigen solution with equal volume of Montanide ISA 51 VG to form a homogeneous emulsion. The AC formulation was prepared by dissolving the peptides and polynucleotide adjuvant in 0.1 M sodium acetate (AC-Survivac).

### Tumour challenge and vaccination

For all tumour challenge experiments, C57BL/6 mice underwent C3 tumour cell implantation, with 5 × 10^5^ cells implanted subcutaneously (s.c.) into the left flank on Study day 0. All vaccine formulations were delivered via a single s.c. contralateral immunization (right flank). Tumour volumes were determined by measurement with calipers and using the following formula: longest measurement × (shortest measurement)2 / 2.

#### DPX vs water-in-oil emulsion

Five days post-implantation (Study Day 0), mice received either i) DPX-R9F: 50 μL of DPX with R9F and F21E (*n* = 23), or ii) w/o-R9F: 100 μL of w/o emulsion with R9F and F21E (*n* = 29). This study was done as 3 separate replicates with *n* = 8 for DPX-R9F groups and *n* = 10 for w/o-R9F (in both groups 1 mouse was eliminated for non-study related health issues).

#### DPX vs other vaccines

Five days post C3 implantation (Study Day 0), mice received either i) DPX-R9F: 50 μL of DPX with R9F and F21E (*n* = 5), ii) Squal o/w: 100 μL of Squal o/w with R9F and F21E (*n* = 7), or iii) Sap/Lip: 100 μL of Sap/Lip with R9F and F21E (*n* = 6). This study was done as one replicate.

### IFN-γ ELISPOT

Mice were implanted with C3 tumours and vaccinated with indicated vaccines on day 5 as described above. Seven or fourteen days after vaccination, mice were euthanized and spleens collected. IFN-γ ELISPOT assay was performed as previously described [10, 17]. Briefly, splenocytes were processed into a single cell suspension and seeded into a 96-well ELISPOT plate coated with anti-IFN-γ at 5 × 10^5^ cells/well (Affymetrix). Cells were stimulated in duplicate with R9F peptide or an irrelevant peptide control (RMFPNAPYL; R9L) at a concentration of 10 μg/mL, or 5 × 10^4^ C3 tumour cells, for background responses no peptide was added. The ELISPOT plate was incubated overnight at 37 °C, 5% CO_2_ and then developed following manufacturers instructions and using AEC substrate kit (Sigma-Aldrich). Spots were counted using an ImmunoSpot Analyzer, ELISPOT plate reader (C.T.L. Ltd., Shaker Heights, OH, U.S.A.) and enumerated as number of spot-forming units (SFU) per well.

### In vitro release of peptide antigens from DepoVax vs. water-in-oil formulation

Dialysis membranes (Spectra/Por^®^, MW cut-off of 250 kDa, Spectrum Laboratories, Rancho Dominigez, CA) were filled with 1 mL of each solution (DPX-Survivac, w/o-Survivac, or AC-Survivac) and immersed in 50 mL of PBS (0.067 M, pH 7.4). The solutions were agitated at 70 rpm on a horizontal shaker with the temperature maintained at 37 ± 2 °C during the experiment. Aliquots of the release media (1 mL) were taken at predetermined time points and replaced with fresh PBS at each time point. The peptide content in the release media was determined by reversed-phase HPLC (RP-HPLC).

### Determination of peptide content by RP-HPLC

Quantification of the synthetic peptide antigens (five survivin peptides and A16L) was performed using a RP-HPLC method. The method used an Agilent 1100 Series HPLC system equipped with a Phenomenex Luna 5 μm C8(2) column. The mobile phase was a gradient of 16–37% (*v*/v) acetonitrile in 0.1% (v/v) aqueous trifluoroacetic acid. Column temperature was maintained at 50 °C, and UV-PDA detection was performed at 215 nm.

### Data acquisition and MR imaging

All data were acquired on a 3 T magnet equipped with 21 cm inner diameter (ID) gradient coil (Magnex Scientific, Oxford, UK) interfaced with a Varian DD Console (Varian Inc., Palo Alto, Ca). A 30 mm ID quadrature transmit/receive RF coil (Doty Scientific, Col., SC) was used to image tumours, vaccination sites, and left & right inguinal lymph nodes simultaneously.

Sagittal images were obtained using a 3D balanced steady-state free precession (bSSFP) sequence with a repetition time (TR)/echo time (TE) = 8/4 ms, flip angle = 30°, a 38.4 × 25.5 × 25.5 mm field of view (FOV) with a 256 × 170 × 170 matrix centred on the torso, giving voxels with 150 μm isotropic resolution. Four signal averages were acquired with four frequencies [22] for a total scan time of approximately 64 min per animal.

For monitoring tumour progression/eradication as well as lymphatic response and antigen clearance, MRI scans were performed weekly for 4–5 weeks. Baseline scans were also performed prior to tumour challenge (Day − 7) to allow proper comparison of anatomical structures.

### MRI image analysis

Volumetric segmentation of structures was performed by a single observer, and was then confirmed by a second independent reviewer. All images were first zero-padded (interpolated to a higher resolution grid to increase the effective resolution and image quality) using ImageJ (NIH). Images were analyzed in RView for each mouse [25, 26]. A semi-automated region growing algorithm was implemented to perform individual 3D segmentations to determine i) C3 tumour volumes, ii) left inguinal lymph node (LLN), iii) right inguinal lymph node volumes (RLN), and the site of injection (SOI).

A semi-quantitative iron mass was calculated for each SOI (see [19] for more details). This semi-quantitative approach estimates injection-site iron mass for inhomogeneous iron distributions within the vaccine depot, by weighting each pixel according to its intensity ratio, and was done for 15, 22, 29 and 36 days post-vaccination (with day 7 normalized to 100%). Statistical significance between groups and across time was initially compared using a two-way ANOVA (*p* < 0.05). Any further differences between groups at each time point were evaluated using student t-tests with Solm-Hidak multiple comparison correction (*p* < 0.05). All statistics were done in GraphPad Prism 6.0 h.

## Results

### DepoVax-based vaccines retain antigens within the oil formulation at the site of injection

**Fig. 1:**
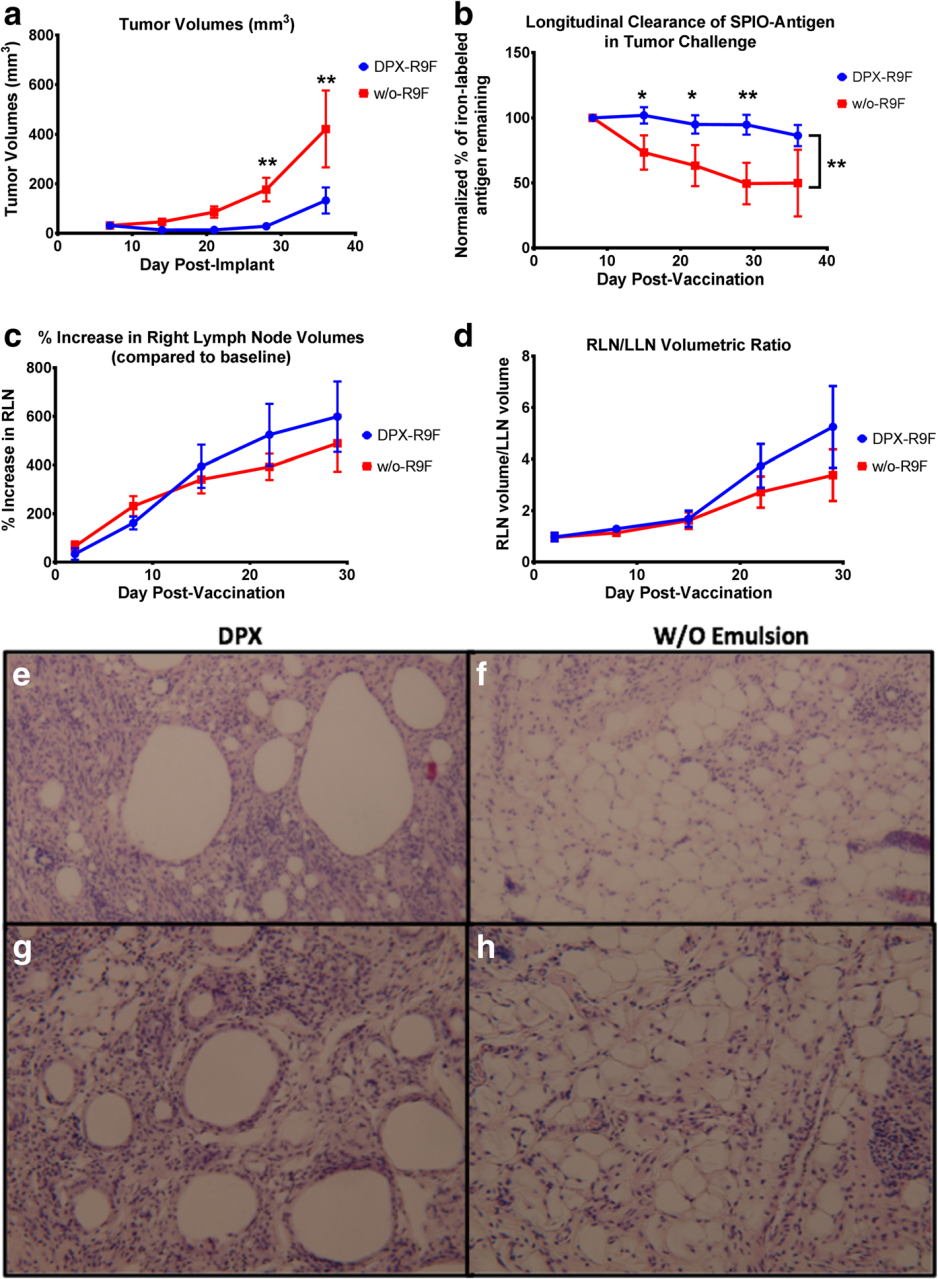
Graphs demonstrating volumetric changes in inguinal lymph nodes and tumours over the course of the study comparing DPX-R9F(*n* = 23) and w/o-R9F (*n* = 29) and Histological Evaluation of Site of Injection for DPX-R9F and w/o-R9F. **a** Tumour volume time course (mm^3^). **b**) Normalized % amount of SPIO-R9F remaining at the SOI. **c** % Right lymph node (RLN) volume increase over time (draining vaccine site). **d** Ratio of RLN volume over LLN volume. Data on graphs is mean ± SE. Significance was calculated using a 2-way ANOVA to compare overall differences across groups and time, and Holm-Sidak corrected t-tests to compare individual time points, * = *p* < 0.1, ** = *p* < 0.05. SOIs were extracted from 5 mice per group 14 days after immunization, sectioned and stained by H&E. Representative figures for 2 different mice are shown for DPX-R9F (**e**, **g**) and w/o-R9F (**f**, **h**)

Immunogenicity of DPX based formulations has been established in preclinical [17, 18] and clinical studies [14, 15]. To compare the efficacy of these vaccines to a w/o emulsion, we performed a tumour challenge study using the C3 tumour model which expresses HPV16 E7. Mice were vaccinated 5 days after implantation with the CD8^+^ T cell epitope HPV16 E7_49–57_ (R9F) formulated in either DPX (DPX-R9F) or water-in-oil emulsion (w/o-R9F). Tumour growth was monitored by imaging as well as external measurement with calipers. As shown in Fig. 1a, the group vaccinated with the DPX-R9F formulation demonstrated better tumour control than w/o-R9F with significantly smaller tumour volumes on day 28 and 35 than the w/o-R9F group (*p* < 0.05).

In our previous work [27, 28], we found that the degree of lymph node swelling induced in the vaccine-draining lymph node (right inguinal lymph node, or RLN) may be a biomarker for the expansion of antigen-specific T cells, and predictive of efficacy in controlling tumour growth. We therefore evaluated the degree of RLN swelling (Fig 1c) and the ratio of the RLN volume to the left inguinal lymph node (LLN) volume (Fig 1d) and found similar degrees of swelling with both vaccines, corresponding with previous work [25, 29]. We evaluated infiltration by histological assessment of H&E stained site of injection (SOI) sections obtained 14 days after immunization (Fig. 1e-h). There is more extensive cellular infiltration in the DPX-R9F (Fig. 1e & g) formulation compared to the w/o-R9F formulation (Fig. 1f & h).

**Fig. 2:**
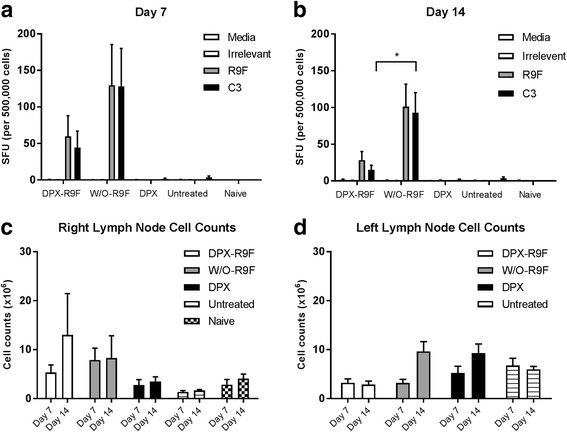
ELISPOT data. Mice (C57BL/6, *n* = 10) were implanted on the left flank with C3 tumours and vaccinated 5 days later with either DPX-R9F, w/o-R9F, DPX (containing no antigen), or untreated. Vaccinations were given subcutaneously on the right flank. Mice (*n* = 5) were terminated 7 days (**a**) and 14 days (**b**) after immunization and IFN-γ ELISPOT performed using splenocytes. Naïve, untreated mice were also included in each experiment. Total lymph node cell counts were performed on the vaccine draining, right inguinal lymph nodes (**c**) and tumour draining left inguinal lymph nodes (**d**) at each time point

Immune responses induced by the vaccines were evaluated using an IFN-γ ELISPOT assay in a parallel study. Mice were terminated on 7 and 14 days after vaccination, spleens as well as vaccine draining (RLN) and contralateral (LLN) inguinal lymph nodes were collected for analysis (Fig. 2). Both DPX-R9F and w/o-R9F vaccines induced R9F-specific immune responses that cross reacted to C3 cells, while the untreated or DPX (vaccine containing no antigen) controls did not. The w/o-R9F vaccine induced stronger immune responses than DPX-R9F at both time points, despite demonstrating less efficacy in controlling tumour volumes.

**Fig. 3:**
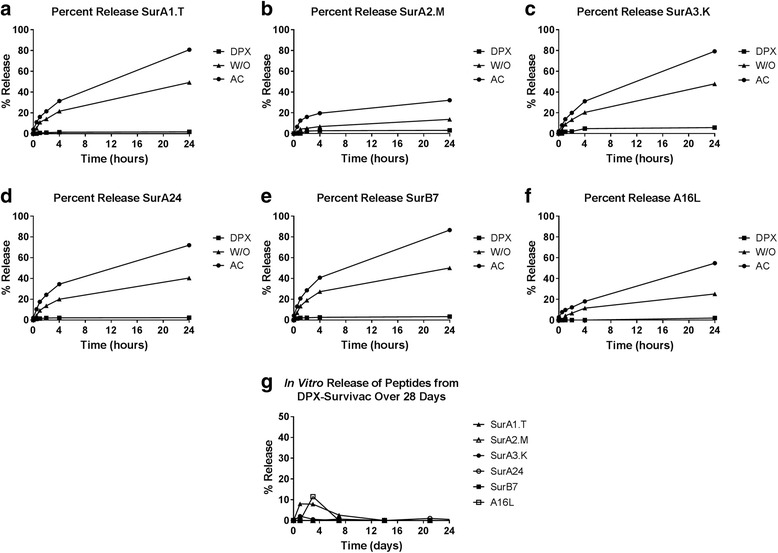
Profiles of peptide release determined by the dialysis tube method comparing DPX-Survivac, w/o-Survivac, and AC-Survivac and evaluating DPX-Survivac over 1 month. Peptide antigens are retained in the DPX formulation when compared with a water-in-oil formulation and an aqueous control. Peptide antigens remain in the DPX formulation. **a**-**g**) Peptide release over 24 h, **h**) Peptide release over 1 month

To compare passive antigen release of these formulations we performed an in vitro dialysis analysis. For this experiment, different vaccine formulations were prepared with 6 peptides used in the clinical product DPX-Survivac. Fig. 3 reports the kinetics observed when dialysis tubes containing DPX-Survivac, w/o-Survivac and AC-Survivac were agitated at 37 °C in PBS. After 4 h, less than 5% of each peptide were detected in the release media of the DPX formulation (Fig. 3a-f). In comparison to the other formulations at the 4 h time point, 7-30%  were recovered from the release media of the w/o formulation and 18-40% were recovered from the release media of the aqueous control.

By 24 h, the percentage of peptides released from the DPX formulation was just under 6%. In contrast, 10–50% of each peptide were released in the w/o- emulsion and 30–80% of were released in the AC formulation. After 1 month, the percentage of peptides that had diffused out of the DPX formulation did not exceed 12% in vitro (Fig. 3g).

**Fig. 4:**
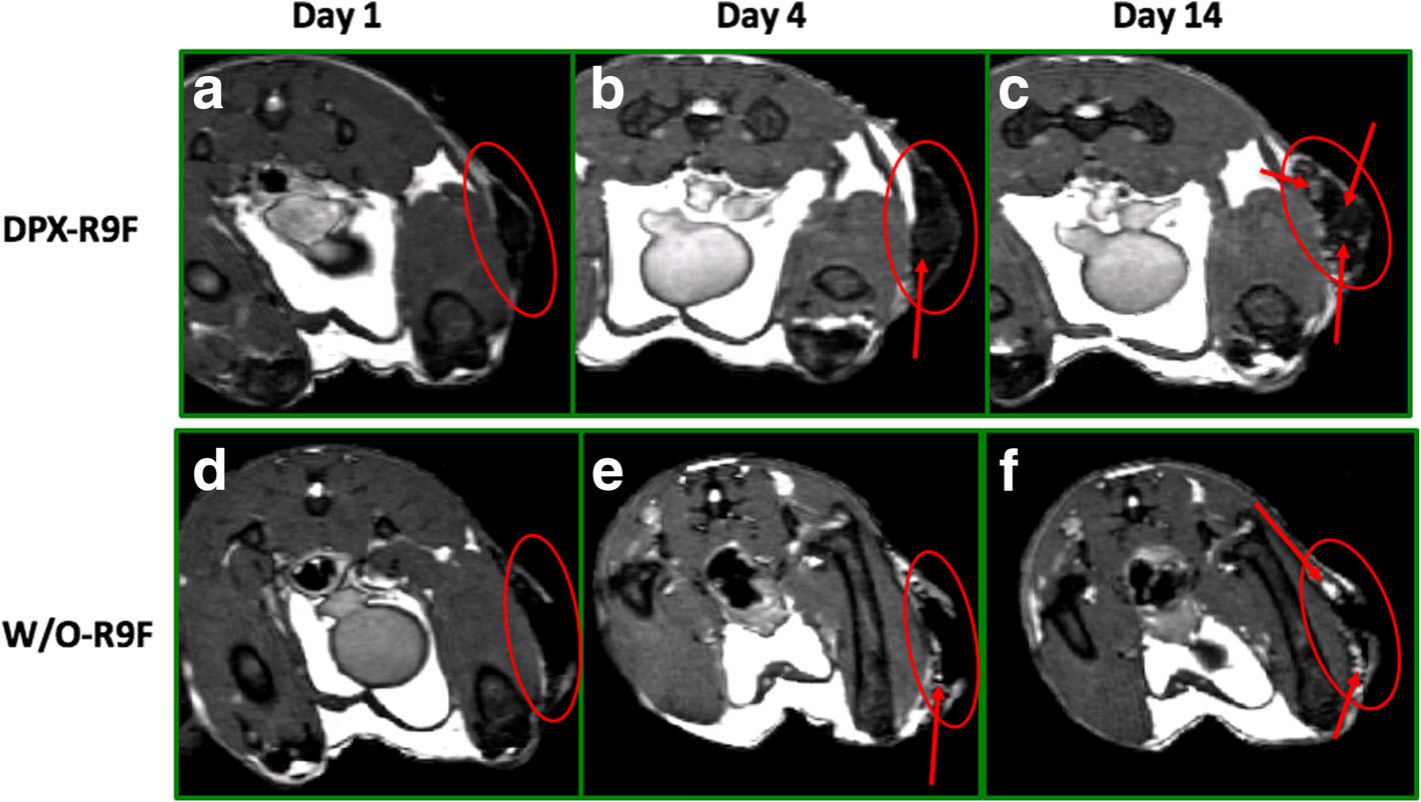
Clearance of Antigen in DPX-R9F and w/o-R9F Detected by MRI. MR images displaying iron content of SPIO-R9F in either **a**), **b**) and **c**) DPX-R9F or d), **e**) and **f**) w/o-emulsion. Images were acquired one (**a** & **d**), four (**b** & **e**), or fourteen (**c** & **f**) days post-immunization. In DPX-R9F SPIO-antigen clears centrally from the vaccine site, from the middle of the site of injection (SOI) outwards, while the SPIO-R9F clears from the edges of the w/o-R9F depot. Red arrows indicate site of active clearance

Peptide release in vivo will be affected by cellular uptake and degradative enzymes in situ, therefore we investigated this further in vivo using MRI to monitor clearance of SPIO-labeled peptides. A semi-quantitative MRI technique was used to monitor iron mass at the vaccine site (described in [14]) with the SPIO-R9F antigen formulated in DPX or w/o-emulsion. Without SPIO, either formulation is visible through MRI pulse sequence bSSFP with bright white signal intensity, due to the presence of the oil-based delivery systems (as seen in [19]). When SPIO is conjugated to R9F, the contrast appears dark or hypointense (Fig. 4), allowing visualization of SPIO clearance over time. The SPIO-R9F clears faster from the w/o injection site compared to the DPX injection site (Fig.1b), and a significant difference was detected on day 28 post injection (Holm-Sidak corrected t-tests, *p* < 0.05) and slightly less significant differences (*p* < 0.1) at days 14 and 21. A two-way ANOVA demonstrated significant differences between both vaccines (*p* < 0.0001) and across time (*p* = 0.0017). Therefore, the DPX formulation has more sustained antigen retention at the site, potentially improving the long term immune response, and extending tumour control. The difference between w/o-R9F and DPX-R9F was not significantly different at day 35, primarily due to higher variability for the w/o emulsion group at that timepoint.

As seen in previous studies [19], the SPIO-R9F clearance from DPX occurs slowly, with a gradual brightening appearing at pockets throughout (Fig. 4a-c), indicating antigen release or removal from more central positions within the vaccine depot. With the w/o emulsion, the signal appears to be brightening quicker at the edges of the SOI (Fig. 4 d-f), indicating that antigen is being released more rapidly from the edges of the vaccine. In our previous work, we demonstrated that the as SPIO-R9F clears from the SOI, it is increasingly detected in the lymph node, indicating active transfer by phagocytic APCs [19]. In comparing the histology of the SOI of these two vaccines (Fig. 1e-h) we found increased immune infiltration of the DepoVax depot compared to the w/o depot. Therefore, the differences in clearance patterns of these vaccines may be correlated to level of immune cell infiltration induced.

### DPX-R9F vs other vaccine types

**Fig. 5:**
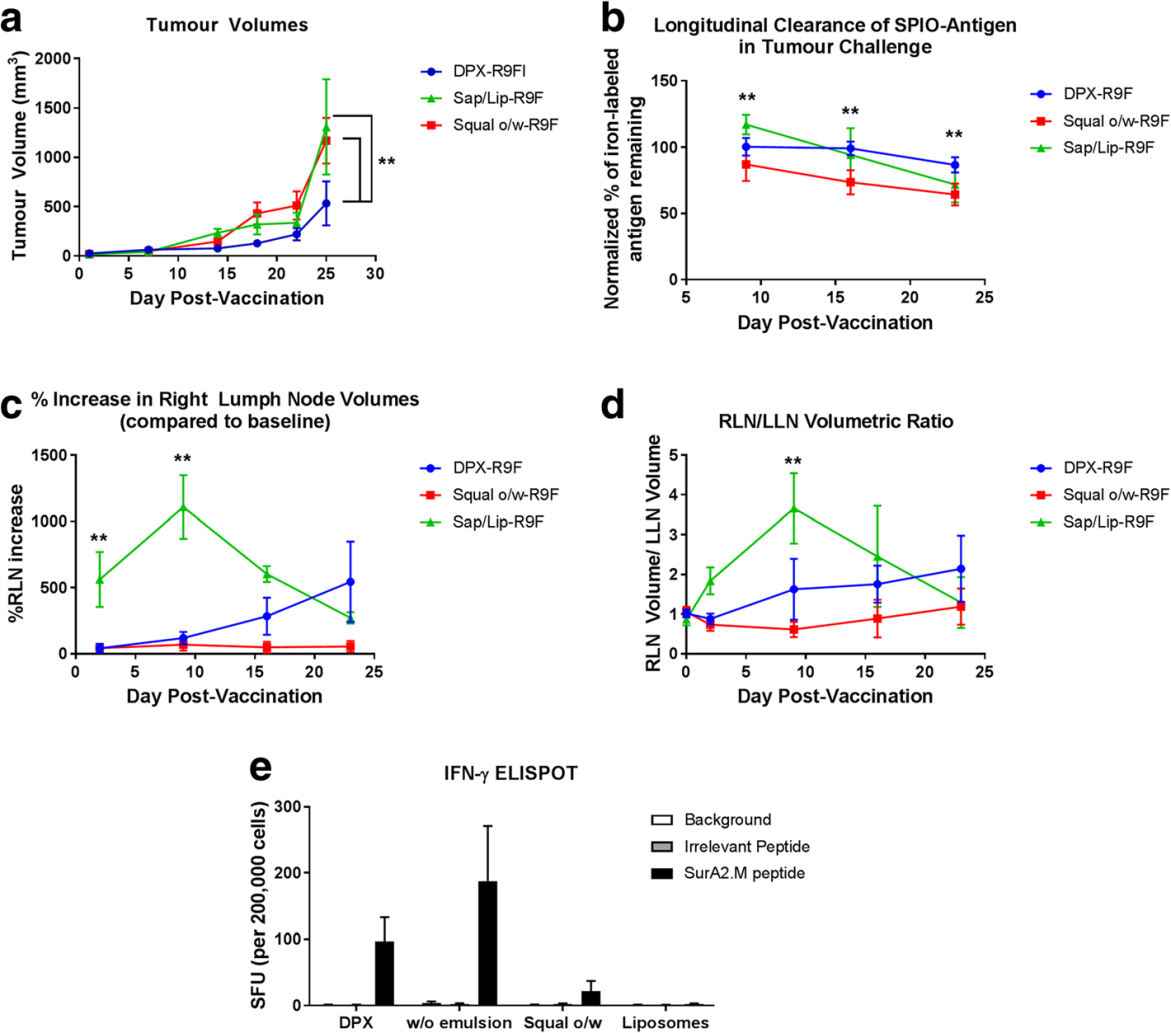
Graphs demonstrating volumetric changes in inguinal lymph nodes and tumours over the course of the study comparing DPX-R9F (*n* = 5) to Squal w/o-R9F (*n* = 7) and Sap/Lip-R9F (*n* = 6) and Immune Response by ELISPOT*.* **a** Tumour volume time course (mm^3^). **b** Normalized % amount SPIO-R9F remaining at the SOI. **c** % Right lymph node (RLN) volume increase over time (draining vaccine site). **d** Ratio of RLN volume over LLN volume. Data on graphs is mean ± SE. Significance was calculated using a 2-way ANOVA to compare overall differences across groups and time, and Holm-Sidak corrected t-tests to compare group differences at individual time points, ** = *p* < 0.05. **e** Immune response by IFN-γ ELISPOT. HLA-A2 transgenic mice (*n* = 5) were vaccinated with a mixture of peptides derived from the survivin protein: SurA1.T, SurA2.M, SurA3.K, SurA24, SurB7 as well as a universal T helper epitope A16L. Peptides were formulated in DepoVax, w/o-emulsion with ISA 51, O/W with squalene, or aqueous liposomes. Eight days after immunization mice were euthanized and spleens removed for IFN-γ ELISPOT. Splenocytes (500,000 cells/ well) were stimulated in duplicate with media alone, an irrelevant peptide (HLA-A2 restricted peptide ALMEQQHYV), or SurA2.M peptide. Results shown as average per group +/− SEM. Statistics by one-way ANOVA comparing responses to SurA2.M stimulation, no statistical significance detected

Although emulsions such as the w/o-R9F are traditionally considered the most common and appropriate comparison for depot-based vaccines like DPX-R9F, a number of other vaccine formulations have been developed to enhance cellular immune responses. We compared DPX-R9F to two other clinically tested vaccine delivery systems: i) a squalene based oil-in-water formulation (squal o/w) and ii) a saponin/liposome (sap/lip) formulation using the C3 challenge model. All formulations contained the R9F peptide antigen labeled with SPIO, and mice were vaccinated 5 days after tumour implantation. Tumour growth kinetics, shown in Fig. 5a, show that the DPX-R9F resulted in significantly better tumour control compared to either of the other formulations (*p* < 0.05).

**Fig. 6:**
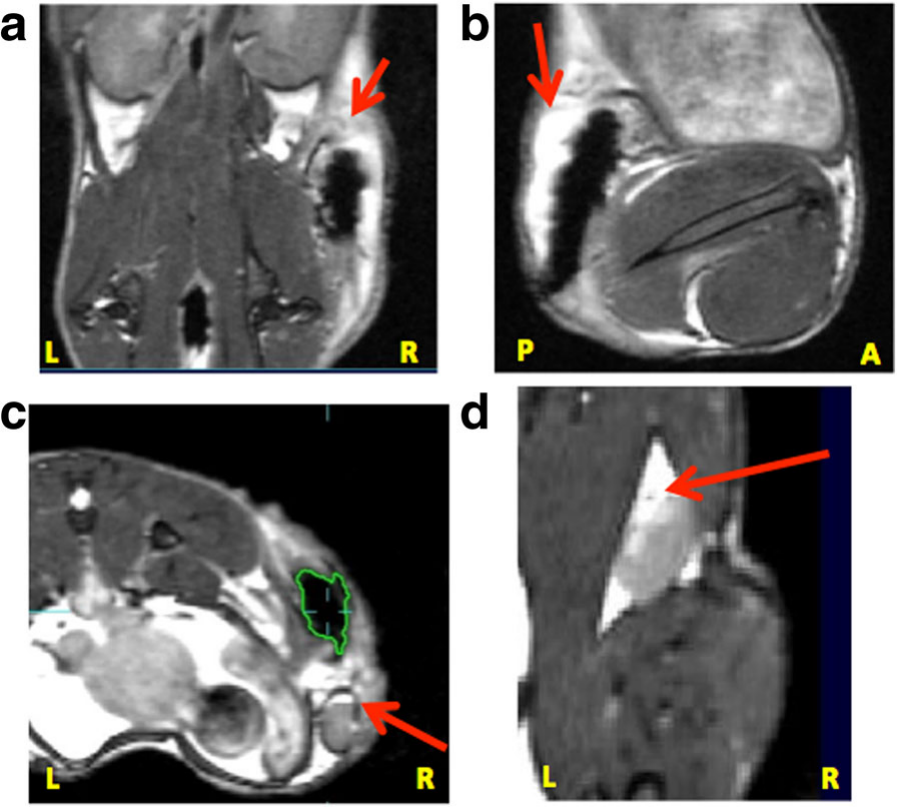
Increased Local Inflammatory Response in Sap/Lip-R9F Vaccine. **a**) BSSFP MRI images (150um)3 isotropic voxels of representative mice (*n* = 2) from the sap/lip-R9F group. Red arrows demonstrate regions with edema as indicated by hyperintensity. Edema can be seen in **a**) and **b**) the SOI, **c**) the vaccine-draining right inguinal lymph node (RLN), and **d**) the right popliteal lymph node

Antigen clearance rates were measured by semi-quantitative iron mass using SPIO labeled R9F antigen (Fig. 5b). There were significant differences between antigen clearance over the 4 weeks of the tumour challenge, with the DepoVax formulation having the most sustained clearance rate. A two-way ANOVA indicated significant differences between both vaccine types (*p* < 0.0001) and across time (*p* < 0.0001). Using Holm-Sidak multiple comparison corrections, on day 9 we detected a significant difference between all three formulations, with the highest SPIO-R9F detected in the sap/lip and the lowest in the squal o/w group. By day 16, the squal o/w water group remained significantly lower than the other formulations, and the sap/lip and DepoVax formulations were not different. By day 23, the DepoVax formulation retained the highest amount of SPIO-R9F, significantly higher than the other two groups (*p* < 0.05). A caveat of this analysis is that accurate quantification of the sap/lip-R9F vaccination at the early time points was challenging due to the presence of extensive edema (as indicated by local regions of signal hyperintensity) surrounding the vaccination site (see Fig. 6a & b). There was considerable swelling at the SOI, which combined with the hyperintensity from the edema, made it extremely difficult to get SOI volumes and signal intensities that accurately represented the presence of iron in the first 2 weeks post-vaccination. By day 23 post-vaccination, the edema and swelling had receded, allowing for more accurate iron quantification. However, since the later time points are compared to the initial amount of iron after vaccination, the iron mass data is likely still somewhat skewed for the sap/lip results. This is a limitation of the semi-quantitative technique for iron mass determination.

The sap/lip-R9F vaccine appeared to cause a visible local reaction at the SOI with two of the five mice in the sap/lip-R9F group needing to be terminated within 1 week of vaccination due to ulceration at the SOI. There was also visible edema in the draining RLN (Fig. 6c) and in some mice, even the right popliteal lymph node. This degree of edema indicates an incredibly strong local inflammatory response. However, the local inflammatory response did not translate to more effective control as all of the sap/lip-R9F vaccinated mice had large tumours present at the end of the study.

The strong local inflammatory response to the sap/lip-R9F vaccine was also visible in the degree of lymph node swelling in the right inguinal lymph node draining the vaccine site (Fig. 5c). The RLN exhibited a statistically significant 500% volumetric increase within 2 days of vaccination (compared to squal o/w, Holm Sidak corrected t-test, *p* < 0.001) and an over 1000% increase within 9 days post-vaccination (Fig. 5c, Holm-Sidak corrected t-test, *p* < 0.005). The degree of RLN swelling in the DPX-R9F group was much more gradual and consistent with previous work demonstrating a gradual response to vaccination. Interestingly, the squal o/w-R9F group did not seem to undergo any swelling in the RLN in response to vaccination, in contrast to all other vaccine formulations (DPX, w/o emulsion and sap/lip).

The large early increase in the RLN also caused a marked increase in the RLN/LLN ratio for the sap/lip-R9F group, with the DPX-R9F group exhibiting similar RLN/LLN growth over the course of the tumour challenge (Fig. 5d). Even the squal o/w-R9F group exhibited increased RLN/LLN at later time points, even though the RLN did not appear to increase. The only significant difference in RLN/LLN ratio between groups was between the squal o/w-R9F vaccine and other vaccine types at day 9. All groups saw increased RLN/LLN ratios later in the tumour challenge, indicating that later time points (i.e. between 16 and 23 days post-vaccination) may be optimal to measure this biomarker to compare different types of vaccines. In this study, individual mice with larger RLN/LLN ratios at approximately 16 days post-vaccination had smaller tumour volumes at the end of the study (although no mice exhibited complete tumour suppression). There was a negative correlation between final tumour volume and RLN/LLN ratio at both day 16 and day 23, but it was only statistically significant at day 23 (Pearson’s correlation − 0.478, *p* < 0.05, one-tailed distribution).

### DPX-Survivac immunogenicity

We compared immune responses induced by these formulations by IFN-γ ELISPOT (Fig. 5e). The sap/lip formulation was excluded in this analysis because it induced significant SOI reactions in the MRI experiments described above, requiring animals be removed from the experiment prior to this time point. Vaccines were prepared using the peptides in the clinical vaccine DPX-Survivac and tested in HLA-A2 transgenic mice. Since these mice only respond to HLA-A2 restricted peptides, we evaluated the immune response to the SurA2.M peptide only. Similar to the ELISPOT performed using vaccines containing the R9F peptide, we found that the w/o-Survivac (average SFU 188) induced stronger antigen-specific immune responses than the DPX-Survivac (average SFU 96) vaccine. The squal o/w-Survivac emulsion however did not generate strong immune responses (average SFU 21).

## Discussion

The results of this study support a unique mechanism through which DepoVax, an oil-based delivery system, activates and maintains persistent antigen-specific T cells in tumour bearing mice. These results contrast DepoVax from emulsion-based depot forming vaccines using real time imaging of the vaccine depot in vivo via MRI.

Cancer vaccines are typically evaluated preclinically based on their ability to induce a T cell based immune response, often limited to single time points due to the requirement to harvest immune organs to complete the assessment. This assessment alone may not be a sufficient predictor of clinical efficacy, particularly considering the complex tumour microenvironment, and the importance of sustained T cell responses in tumour control. Our study demonstrates that clearance rate and reactogenicity can impact the overall efficacy of a vaccine; factors that can be controlled with an appropriate delivery system. In this study, the depot formed by the w/o-emulsion allowed for relatively rapid release from the SOI, likely due to a breakdown of the emulsion in vivo, releasing the vaccine components held in the aqueous phase. The lack of immune cells detected in the histology sections and the results of the in vitro dialysis experiment support the hypothesis that this is a passive release into the surrounding tissue. Ultimately, although this vaccine consistently induced a stronger T cell response at a single time point when compared to DPX formulations, it was less effective overall in controlling tumour growth. In preclinical immunogenicity studies of DPX-Survivac, we have detected antigen specific immune responses by IFN-γ ELISPOT up to 50 days after a single immunization supporting the sustained ability to present peptides and facilitate T cell responses (Additional file 1: Figure S1). In this study, we have used a subcutaneous tumour model, the C3 model, to demonstrate efficacy. Although this model is not orthotopic, it is useful for MRI imaging since the tumours can be consistently resolved and immune responses to the R9F antigen used in the vaccine correlate with tumour control. Orthotopic models are currently being developed for future invetigations.

In preclinical studies, it has been shown that emulsion based formulations can attract circulating T cells back to the injection site, reducing circulating antigen-specific T cells and increasing immune suppression [9, 16]. This reduction in circulating antigen-specific T cells has not been demonstrated in clinical trials with DPX-Survivac, where both *ex vivo* ELISPOT analysis and multimer staining of PBMCs has detected antigen-specific T cells 168 days after the last immunization [14, 15]. The other emulsion type vaccines tested in this study either induced strong inflammatory responses or failed to control tumour growth. In contrast, DPX has no aqueous component, and the clearance from the SOI coupled with the accumulation of SPIO signal in the draining lymph nodes indicates that the vaccine is likely actively engulfed by APCs. This formulation containing both antigen and adjuvant, can facilitate persistent interaction with the immune system. The DPX formulation consistently provided the most effective control of tumour growth, supporting the superiority of the formulation in inducing T cells effective in controlling tumours.

The in vitro release study demonstrates that the DPX formulation does not support passive release of antigens, unlike the emulsion formulation. The hydrophobic reverse micelles in the DPX formulation hold the vaccine components within a stable oil phase and limit interaction with the surrounding aqueous buffer [30, 31]. In vivo, peptide release can occur passively and actively, through phagocytosis by antigen presenting cells. In our previous work, we show that the gradual clearance of the antigen from the SOI correlates to gradual increase in antigen at the lymph node. The histology of the SOI in this work (Fig. 1) shows a higher level of immune infiltration of the DPX formulation, supporting the hypothesis that peptide clearance from the SOI is an active process. The clearance patterns of SPIO-R9F also support that the DPX formulation is cleared primarily through active uptake rather than passive (Fig. 4). Although the in vivo immune responses generated by DPX, as detected by IFN-γ ELISPOT, were not higher than other formulations, DPX consistently provided better tumour protection than any of the emulsion based delivery systems it was compared to. This unique presentation of antigen and adjuvant to the immune system may prevent suppressive immune responses developing alongside effector immune response, as we have previously observed when comparing a DPX formulation to an emulsion [16].

## Conclusions

This work uses a combination of imaging, immunogenicity and pathology to demonstrate that the DepoVax-based formulation is unique in its presentation of antigens to the immune system when compared to emulsion-based vaccines. DepoVax facilitates the development and persistence of antigen-specific T cells in tumour bearing mice, consistent with data using this vaccine formulation in advanced cancer patients. This type of formulation may be uniquely poised to generate strong and sustained T cell responses that are linked to clinical benefit in cancer immunotherapies.

## Additional file

Additional file 1: Figure S1. Immune responses of DPX-formulated vaccine up to 50 days. HLA-A2 transgenic mice (HHD-DR1) received a single subcutaneous immunization with 50 uL of DPX-Survivac in the right flank. Groups of mice (*n* = 5) were terminated 8, 22 and 50 days after immunization and IFN-γ ELISPOT performed using lymph node cells isolated from the right inguinal lymph node. Cells were stimulated with syngeneic dendritic cells loaded with no peptide (empty), irrelevant peptide (ALMEQQHYV), or SurA2.M (LMLGEFLKL). (DOCX 40 kb)

## Abbreviations

AC: Aqueous Control
ANOVA: Analysis of Variance
DC: Dendritic cells
DOPC: 1,2-dioleoyl-sn-glycero-3-phosphocholine
DPX: DepoVax™
ID: Inner diameter
LLN: Left lymph node
LN: Lymph node
MRI: Magnetic Resonance Imaging
RLN: Right lymph node
ROI: Region of Interest
Sap/Lip: Saponin/liposome
Squal o/w: Squalene oil-in-water formulation
w/o emulsion: Water-in-oil emulsion
w/o: Water-in-oil

## References

[1] Palucka AK, Coussens LM (2016). The basis of Oncoimmunology. Cell.

[2] van der Burg SH, Arens R, Ossendorp F, van Hall T, Melief CJ (2016). Vaccines for established cancer: overcoming the challenges posed by immune evasion. Nat Rev Cancer.

[3] Gilbert SC (2012). T-cell-inducing vaccines - what's the future. Immunology.

[4] Slingluff CL, Lee S, Zhao F, Chianese-Bullock KA, Olson WC, Butterfield LH, Whiteside TL, Leming PD, Kirkwood JM (2013). A randomized phase II trial of multiepitope vaccination with melanoma peptides for cytotoxic T cells and helper T cells for patients with metastatic melanoma (E1602). Clin Cancer Res.

[5] Khong H, Overwijk WW (2016). Adjuvants for peptide-based cancer vaccines. J Immunother Cancer.

[6] Bouvier I, Jusforgues-Saklani H, Lim A, Lemaitre F, Lemercier B, Auriau C, Nicola MA, Leroy S, Law HK, Bandeira A (2011). Immunization route dictates cross-priming efficiency and impacts the optimal timing of adjuvant delivery. Front Immunol.

[7] Rosenberg SA, Yang JC, Restifo NP (2004). Cancer immunotherapy: moving beyond current vaccines. Nat Med.

[8] Suzuki N, Hazama S, Iguchi H, Uesugi K, Tanaka H, Hirakawa K, Aruga A, Hatori T, Ishizaki H, Umeda Y (2017). Phase II clinical trial of peptide cocktail therapy for patients with advanced pancreatic cancer: VENUS-PC study. Cancer Sci.

[9] Hailemichael Y, Dai Z, Jaffarzad N, Ye Y, Medina MA, Huang XF, Dorta-Estremera SM, Greeley NR, Nitti G, Peng W (2013). Persistent antigen at vaccination sites induces tumor-specific CD8(+) T cell sequestration, dysfunction and deletion. Nat Med.

[10] Salerno EP, Shea SM, Olson WC, Petroni GR, Smolkin ME, McSkimming C, Chianese-Bullock KA, Slingluff CL (2013). Activation, dysfunction and retention of T cells in vaccine sites after injection of incomplete Freund's adjuvant, with or without peptide. Cancer Immunol Immunother.

[11] Petrovsky N (2015). Comparative safety of vaccine Adjuvants: a summary of current evidence and future needs. Drug Saf.

[12] de Vos van Steenwijk PJ, van Poelgeest MI, Ramwadhdoebe TH, Lowik MJ, Berends-van der Meer DM, van der Minne CE, Loof NM, Stynenbosch LF, Fathers LM, Valentijn AR (2014). The long-term immune response after HPV16 peptide vaccination in women with low-grade pre-malignant disorders of the uterine cervix: a placebo-controlled phase II study. Cancer Immunol Immunother.

[13] Temizoz B, Kuroda E, Ishii KJ (2016). Vaccine adjuvants as potential cancer immunotherapeutics. Int Immunol.

[14] Berinstein NL, Karkada M, Morse MA, Nemunaitis JJ, Chatta G, Kaufman H, Odunsi K, Nigam R, Sammatur L, MacDonald LD (2012). First-in-man application of a novel therapeutic cancer vaccine formulation with the capacity to induce multi-functional T cell responses in ovarian, breast and prostate cancer patients. J Transl Med.

[15] Berinstein NL, Karkada M, Oza AM, Odunsi K, Villella JA, Nemunaitis JJ, Morse MA, Pejovic T, Bentley J, Buyse M (2015). Survivin-targeted immunotherapy drives robust polyfunctional T cell generation and differentiation in advanced ovarian cancer patients. Oncoimmunology.

[16] Karkada M, Weir GM, Quinton T, Sammatur L, MacDonald LD, Grant A, Liwski R, Juskevicius R, Sinnathamby G, Philip R, Mansour M (2010). A novel breast/ovarian cancer peptide vaccine platform that promotes specific type-1 but not Treg/Tr1-type responses. J Immunother.

[17] Weir GM, Hrytsenko O, Stanford MM, Berinstein NL, Karkada M, Liwski RS, Mansour M (2014). Metronomic cyclophosphamide enhances HPV16E7 peptide vaccine induced antigen-specific and cytotoxic T-cell mediated antitumor immune response. Oncoimmunology.

[18] Weir GM, Hrytsenko O, Quinton T, Berinstein NL, Stanford MM, Mansour M (2016). Anti-PD-1 increases the clonality and activity of tumor infiltrating antigen specific T cells induced by a potent immune therapy consisting of vaccine and metronomic cyclophosphamide. J Immunother Cancer.

[19] Brewer KD, Lake K, Pelot N, Stanford MM, DeBay DR, Penwell A, Weir GM, Karkada M, Mansour M, Bowen CV (2014). Clearance of depot vaccine SPIO-labeled antigen and substrate visualized using MRI. Vaccine.

[20] Feltkamp MC, Smits HL, Vierboom MP, Minnaar RP, de Jongh BM, Drijfhout JW, ter Schegget J, Melief CJ, Kast WM (1993). Vaccination with cytotoxic T lymphocyte epitope-containing peptide protects against a tumor induced by human papillomavirus type 16-transformed cells. Eur J Immunol.

[21] Smith KA, Meisenburg BL, Tam VL, Pagarigan RR, Wong R, Joea DK, Lantzy L, Carrillo MA, Gross TM, Malyankar UM (2009). Lymph node-targeted immunotherapy mediates potent immunity resulting in regression of isolated or metastatic human papillomavirus-transformed tumors. Clin Cancer Res.

[22] Aucouturier J, Dupuis L, Ganne V (2001). Adjuvants designed for veterinary and human vaccines. Vaccine.

[23] Calabro S, Tortoli M, Baudner BC, Pacitto A, Cortese M, O'Hagan DT, De Gregorio E, Seubert A, Wack A (2011). Vaccine adjuvants alum and MF59 induce rapid recruitment of neutrophils and monocytes that participate in antigen transport to draining lymph nodes. Vaccine.

[24] Alving CR, Peachman KK, Rao M, Reed SG (2012). Adjuvants for human vaccines. Curr Opin Immunol.

[25] Studholme C. Rview Software n.d. 2016.

[26] Studholme C, Hill DLG, Hawkes DJ (1999). An overlap invariant entropy measure of 3D medical image alignment. Pattern Recogn.

[27] Brewer KD, DeBay DR, Dude I, Davis C, Lake K, Parsons C, Rajagopalan R, Weir G, Stanford MM, Mansour M, Bowen CV (2016). Using lymph node swelling as a potential biomarker for successful vaccination. Oncotarget.

[28] DeBay DR, Brewer KD, LeBlanc SA, Weir GM, Stanford MM, Mansour M, Bowen CV (2015). Using MRI to evaluate and predict therapeutic success from depot-based cancer vaccines. Mol Ther Methods Clin Dev.

[29] Scheffler K (2003). On the transient phase of balanced SSFP sequences. Magn Reson Med.

[30] Jones MC, Tewari P, Blei C, Hales K, Pochan DJ, Leroux JC (2006). Self-assembled nanocages for hydrophilic guest molecules. J Am Chem Soc.

[31] Kranz H, Bodmeier R (2007). A novel in situ forming drug delivery system for controlled parenteral drug delivery. Int J Pharm.

