# Supplementary material for: Unique depot formed by an oil based vaccine facilitates active antigen uptake and provides effective tumour control
Source: J Biomed Sci. 2018 Jan 27;25:7. doi: 10.1186/s12929-018-0413-9 (PMC5787234; doi:10.1186/s12929-018-0413-9)
Supplement: Additional file 1: Figure S1. — Immune responses of DPX-formulated vaccine up to 50 days. HLA-A2 transgenic mice (HHD-DR1) received a single subcutaneous immunization with 50 uL of DPX-Survivac in the right flank. Groups of mice (n = 5) were terminated 8, 22 and 50 days after immunization and IFN-γ ELISPOT performed using lymph node cells isolated from the right inguinal lymph node. Cells were stimulated with syngeneic dendritic cells loaded with no peptide (empty), irrelevant peptide (ALMEQQHYV), or SurA2.M (LMLGEFLKL). (DOCX 40 kb) [file 12929_2018_413_MOESM1_ESM.docx]

Supplement Figure 1: Immune responses of DPX-formulated vaccine up to 50 days. HLA-A2 transgenic mice (HHD-DR1) received a single subcutaneous immunization with 50 uL of DPX-Survivac in the right flank. Groups of mice (n=5) were terminated 8, 22 and 50 days after immunization and IFN-g ELISPOT performed using lymph node cells isolated from the right inguinal lymph node. Cells were stimulated with syngeneic dendritic cells loaded with no peptide (empty), irrelevant peptide (), or SurA2.M (LMLGEFLKL).
